# Unusual Symbiotic Cyanobacteria Association in the Genetically Diverse Intertidal Marine Sponge *Hymeniacidon perlevis* (Demospongiae, Halichondrida)

**DOI:** 10.1371/journal.pone.0051834

**Published:** 2012-12-14

**Authors:** Anoop Alex, Vitor Vasconcelos, Paula Tamagnini, Arlete Santos, Agostinho Antunes

**Affiliations:** 1 CIMAR/CIIMAR, Centro Interdisciplinar de Investigação Marinha e Ambiental, Universidade do Porto, Porto, Portugal; 2 Departamento de Biologia, Faculdade de Ciências, Universidade do Porto, Porto, Portugal; 3 IBMC- Instituto de Biologia Molecular e Celular, Universidade do Porto, Porto, Portugal; Columbia University, United States of America

## Abstract

Cyanobacteria represent one of the most common members of the sponge-associated bacterial community and are abundant symbionts of coral reef ecosystems. In this study we used Transmission Electron Microscopy (TEM) and molecular techniques (16S rRNA gene marker) to characterize the spatial distribution of cyanobionts in the widely dispersed marine intertidal sponge *Hymeniacidon perlevis* along the coast of Portugal (Atlantic Ocean). We described new sponge associated cyanobacterial morphotypes (*Xenococcus*-like) and we further observed *Acaryochloris* sp. as a sponge symbiont, previously only reported in association with ascidians. Besides these two unique cyanobacteria, *H*. *perlevis* predominantly harbored *Synechococcus* sp. and uncultured marine cyanobacteria. Our study supports the hypothesis that the community of sponge cyanobionts varies irrespective of the geographical location and is likely influenced by seasonal fluctuations. The observed multiple cyanobacterial association among sponges of the same host species over a large distance may be attributed to horizontal transfer of symbionts. This may explain the absence of a co-evolutionary pattern between the sponge host and its symbionts. Finally, in spite of the short geographic sampling distance covered, we observed an unexpected high intra-specific genetic diversity in *H*. *perlevis* using the mitochondrial genes ATP6 (π = 0.00177), COI (π = 0.00241) and intergenic spacer SP1 (π = 0.00277) relative to the levels of genetic variation of marine sponges elsewhere. Our study suggests that genotypic variation among the sponge host *H*. *perlevis* and the associated symbiotic cyanobacteria diversity may be larger than previously recognized.

## Introduction

The symbiotic association of prokaryotes and eukaryotes, which involves a partnership between the host and partner, has been well documented in several lineages of plants and animals [1], [2], [3] from diverse ecosystems [4]. Symbiont-host partnership is common among marine multicellular sessile organisms such as sponges [5], cnidarians [6], and ascidians [7], because of their limited ability to procure essential nutrients directly and the relative difficulty and the cost to the symbiont to colonize moving partners.

Sponges (phylum Porifera), the most ancient multicellular filter feeder animals, host a wide range of symbiotic microorganisms that have been largely represented by both heterotrophic and photosynthetic bacteria [8] since the Precambrian age [9]. The body plan of this basal metazoan, with a large surface area to volume and the ability to filter feed, provided numerous possibilities for diverse microorganisms to evade the sponge immune system and be housed in the sponge host [10]. Nevertheless, the cellular localization of symbiotic microbes depends on the type of symbionts and the niche-specific habitat of the host partner. These symbiotic associations could influence the potential growth of the sponge host (directly or indirectly) and interfere with morphological plasticity and behavior of the host [11].

Although the nature of symbiosis is not well understood, previous evidence suggests that photobionts have been a significant driving force in the evolution of their hosts [12]. Cyanobacteria, the photosynthetic symbionts are common among temperate and tropical coral reef sponges [13] apart from Zooxanthellae and filamentous algae [14]. Symbiotic cyanobacteria provide a range of specialized services for the host’s survival and growth, including photosynthesis, nitrogen fixation [15], UV protection [16], [17], and antifeedants [18]. Cyanobionts contribute up to 80% of sponge’s carbon budget [19] through photosynthesis or phagocytosis and digestion of symbiotic microbes [20].

Coral reef sponges have been reported to be colonized by cyanobacterial symbionts belonging to the genera *Synechocystis* [21], *Aphanocapsa* [22], [23], and *Anabena* [24] and the species *Ocillatoria spongeliae* [25], [26], [27]. The most common sponge cyanobiont, *Candidatus Synechococcus spongiarum*, has a generalist to specialist association-pattern across distantly related host species despite their geographical isolation by distance [28], which has been hypothesized to be the result of selective enrichment by the host [29]. Cyanobacterial symbionts may be acquired either vertically, from parent to offspring (through larvae) or horizontally, from the surrounding environment. Larval transmission of symbionts is believed to give immediate access of nutrients for the host [30]. Horizontal transmission occurs when the host acquires novel symbionts that are adapted to local conditions [31].

Apart from symbiotic variation, understanding the population genetic structure of the host is a significant aspect of sponge-symbiont evolution. The genetic structure of marine organisms has been inferred using the maternally-inherited mitochondrial gene cytochrome oxidase I (COI) [32], for example in the sponges *Callyspongia vaginalis* [33] and *Cliona celata* [34]. The COI marker has been also used to assess the phylogenetic relationships among other sponge orders [35].

**Figure 1:**
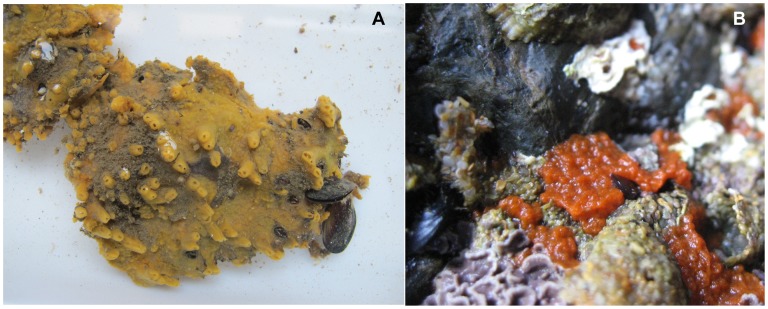
Image of *Hymeniacidon perlevis* collected from two different intertidal rocky shores (Praia da Memória, Praia de Porto Côvo) in Portugal. (A) Encrusting orange-yellow sponge, surface covered with irregularly shaped long/short papillae ranging from 1–3 cm and oscular chimneys. (B) Deep blood-red color with slimy appearance in the natural habitat.

Here, we studied *Hymeniacidon perlevis* (Montagu, 1818), an intertidal sponge in the Halichondriidae family that is exposed to sun during low tide and which has a greater likelihood of harboring cyanobacteria compared with shallow reef sponges. *Hymeniacidon perlevis* are found from the Atlantic coast of Europe to the Mediterranean and Canary Island in habitats ranging from rocky intertidal to shallow subtidal [36]. The species has also been found recently in the Yellow Sea, China [37], [38], [39]. Live specimens of *H. perlevis* have distinct color patterns ranging from orange to blood-red [40] depending on different geographical location (Figure 1).

The aim of this study was to compare the spatial distribution of cyanobacterial symbionts among the host sponge *H. perlevis* at different geographical intertidal locations along the Atlantic coast of Portugal. Host associated cyanobacteria were determined based on a combined strategy that included Transmission Electron Microscopy (TEM) and molecular genetics species identification using the 16S rRNA gene (400–920 bp). We identified two previously undescribed associated cyanobacteria in *H. perlevis* from different sampling locations (*Xenococcus*-like morphotypes and *Acaryochloris* sp.). Other common sponge associated cyanobacteria, including *Synechococcus* sp. and uncultured marine cyanobacteria, were also found in the sponge host. Finally, the genetic assessment of *H*. *perlevis* using the COI, the ATP synthase subunit 6 (ATP6) and the intergenic spacer region (SP1) revealed considerable intra-specific genetic variation within a short geographical distance (∼500 Km), in contrast with previous sponges genetic studies that described low genetic variability at larger geographic scales.

## Results

### Cyanobionts in *Hymeniacidon perlevis*: Transmission Electron Microscopy and Phylogenetic Analyses

Sampling along the Atlantic coast of Portugal identified multiple cyanobionts within the sponge host *H*. *perlevis*, consisting primarily of 47% *Synechococcus*, 42% uncultured cyanobacteria and two unique cyanobacteria, *Xenococcus*-like morphotypes and *Acaryochloris* sp. We observed a generalist association of cyanobacteria in sponges at different climatic conditions and within the same geographical locations, particularly in Praia de Porto Côvo.

**Figure 2:**
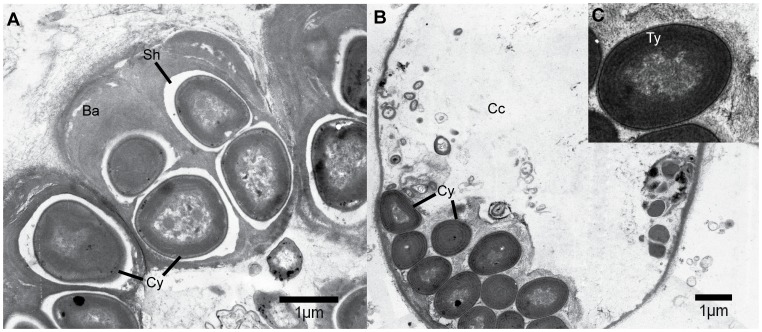
Transmission electron microscopy of cyanobionts from the sponge *Hymeniacidon perlevis* (Sponge ID HYM5B). (A) *Xenococcus* like morphotypes observed at dividing stage with prominent baeocytes with gelatinous outer sheath. (B) Cyanobacterial colony showing compactly packed spiral thylakoids in choanocyte chamber. (C) Insight showing zoomed in image of cyanobacterial cell. (Cy, Cyanobacteria; Ba, Baeocytes; Sh, Sheath; Cc, choanocyte chamber; Ty, Thylakoid).

Transmission electron microscopy on the thin sections of sponge tissue (sponge ID-HYM5B) revealed the presence of unicellular coccoid cyanobacteria *Xenococcus*-like morphotypes in the choanocyte chambers (Figure 2A). The cyanobacterial cells were irregular rounded to rounded-polygonal in shape, with a diameter of 1 to 1.5 µm. Some cyanobacterial cells were at dividing stage, releasing baeocytes with prominent outer sheath/capsule. The observed symbiont had a smooth cell wall and compactly packed six-to ten-spiral thylakoids (Figure 2B, 2C).

The partial 16S rRNA sequence from sponge ID-HYM5B showed 94% similarity (0.0 E-value) to *Xenococcus* sp. PCC 7305. BLAST search also retrieved similarity with the cyanobacteria *Cyanotheca* sp., *Solentia* sp., uncultured cyanobacteria, *Aphanocapsa* sp., *Gloeocapsa* sp. and the symbiont of *Climacodium frauenfeldianum* with 94% similarity and 0.0 E-value. Comparison of partial 16S rRNA gene sequences (from other samples) using BLAST recovered *Synechococcus* sp. (nine specimens) and uncultured marine cyanobacteria (eight specimens) as the best hits. The sequence obtained from sponge ID-HYM16B shared similarity with *Acaryochloris* sp. Awaji-1 partial 16S rRNA gene (100% coverage, 92% max. identity) and *Synechococcus* sp. PCC 7001 (99% coverage, 92% max. identity).

**Figure 3:**
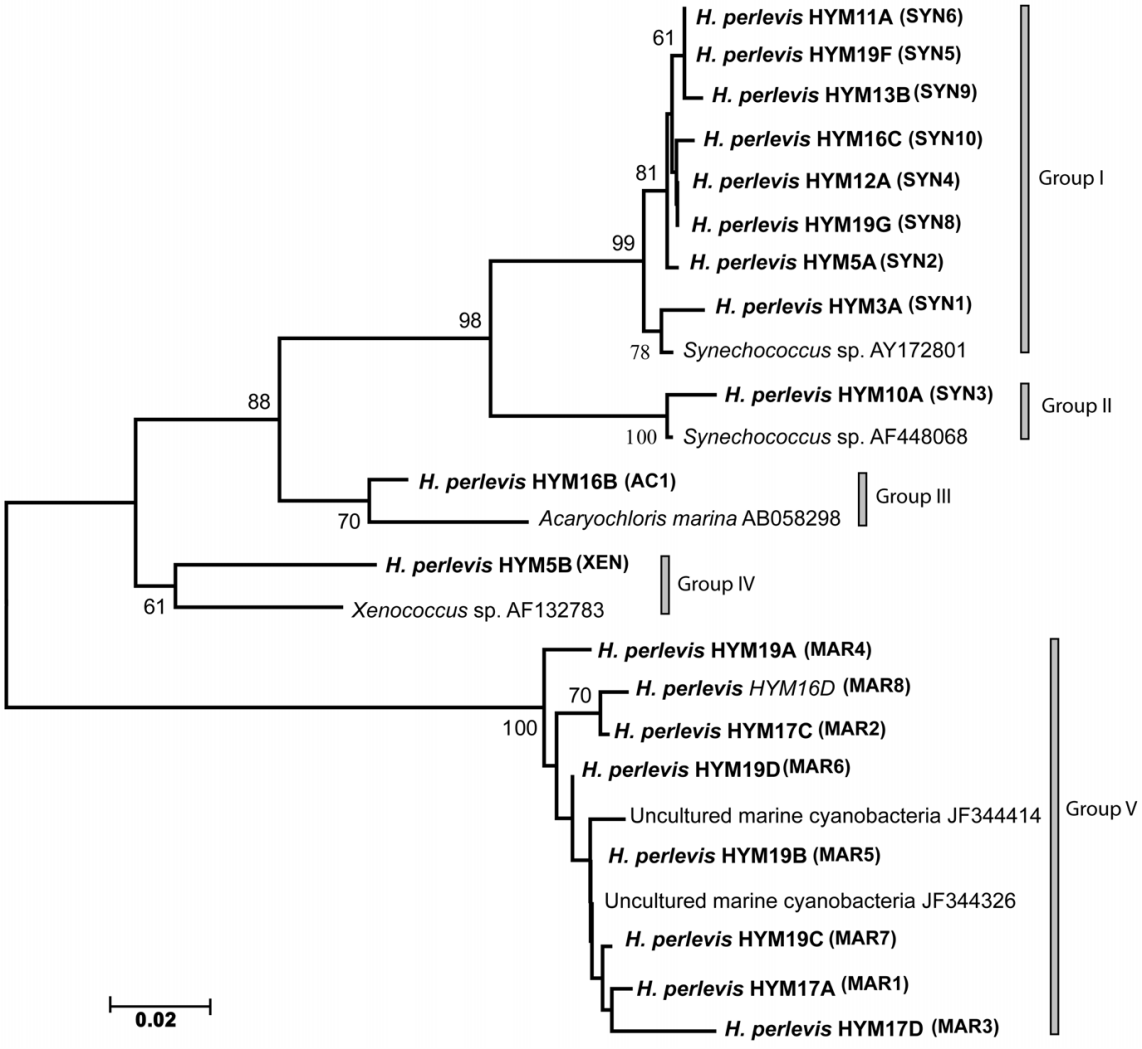
Sponge associated cyanobacterial 16S rRNA neighbor-joining tree. Sponge host, *H. perlevis* associated cyanobacteria inferred from 19 specimens are represented in bold by its host name following the specimen ID and identified cyanobacteria with respective ID in parentheses, which are represented by five groups. The group I and group II represents *Synechococcus* sp., group III constitute *Acaryochloris* sp., group IV represents *Xenococcus* sp. and uncultured cyanobacteria in group V. Bootstrap values above 50% are indicated. Scale bar represents 0.02 substitutions per site. (See Table S1 for sponge specimen ID, inferred cyanobionts ID and collection site).

**Figure 4:**
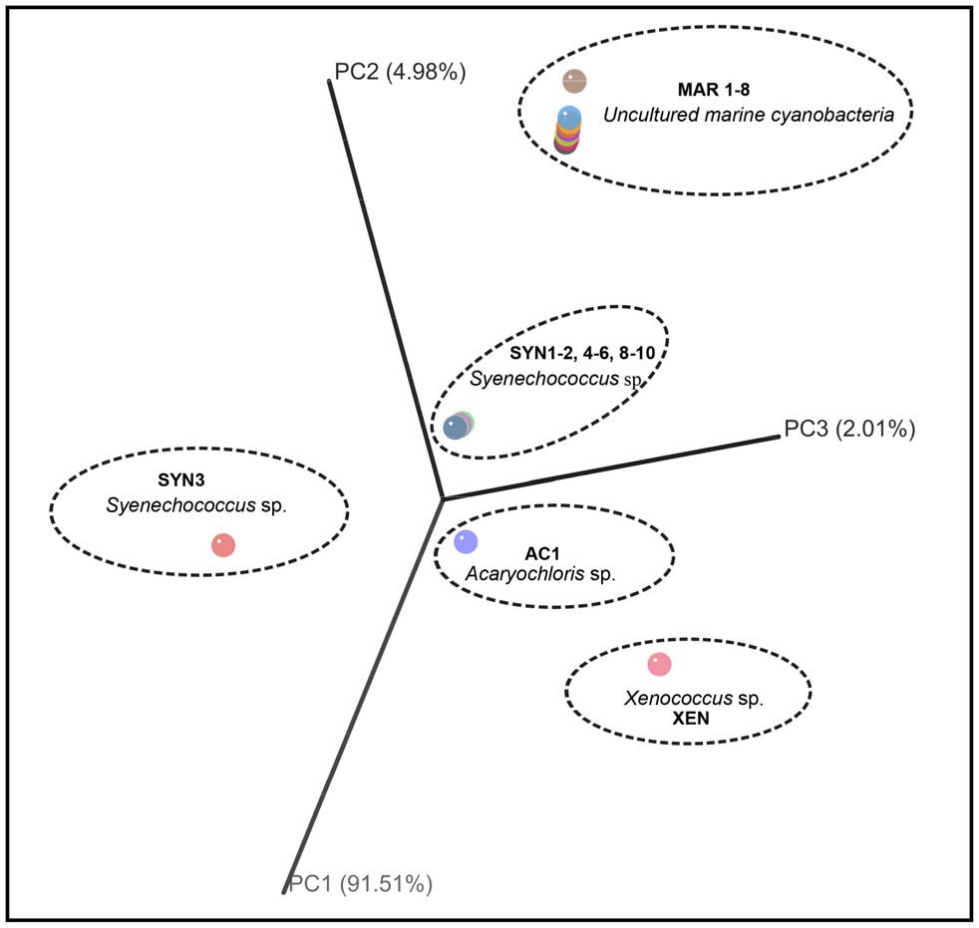
Principal coordinates analysis (PCoA) of cyanobacteria associated with 19 specimens of the sponge host *H. perlevis*. The plot was constructed with FastUniFrac web server using NJ tree inferred from 16S rRNA gene dataset (see materials and methods). Elliptical dashed line represents group of distinct cyanobacterial community with its ID in bold letters- *Synechococcus* sp. (SYN), *Acaryochloris* sp. (AC), *Xenococcus* sp. (XEN), uncultured marine cyanobacteria (MAR) from the sponge specimens. (See Table S1 for sponge specimen ID and inferred cyanobionts ID in detail).

**Figure 5:**
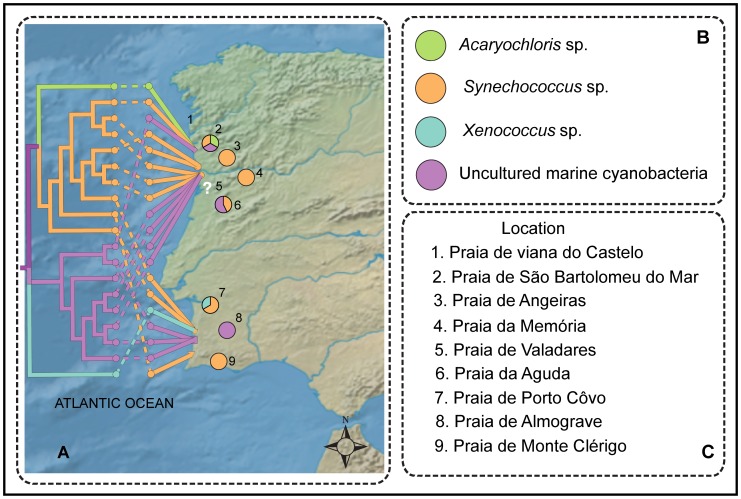
Geophylogeny of *H*. *perlevis* and the cyanobionts. (A) Neighbor-joining tree using 16S rRNA gene of symbiotic cyanobacteria merged with geographical data. Evolutionary distances were calculated using p-distance. Leaf node color represents associated symbionts connected with the geographical line through respective dashed correlation line. Pie chart represents the frequency of symbionts from each location. White question mark denotes the locations where sponge associated cyanobacteria could not be identified. (B) The color representation for each symbiont. (C) Location number used in map and respective site.

The Neighbor-Joining phylogenetic analysis of the 16S rRNA gene inferred from 19 sponge-associated cyanobacteria clustered as five distinct sponge-associated cyanobacterial groups, *Synechococcus* sp. (group I, II, originated from sponge HYM3A, HYM5A, HYM10A, HYM11A, HYM12A, HYM13A, HYM13B, HYM19F, HYM19G), *Acaryochloris* sp. (group III, HYM16B), *Xenococcu*s sp. (group IV, HYM5B) and uncultured marine cyanobacteria in group V (sponge ID- HYM16D, HYM17A, HYM17C, HYM17D, HYM19A, HYM19B, HYM19C, HYM19D) (Figure 3). PCoA analysis supported the distinct clustering of the cyanobacteria (Figure 4). Geophylogeny [41] suggested that the distribution pattern of cyanobacterial symbionts from the sponge sampled at different geographical coordinates was spatially inconsistent, harboring multiple partners within the sponge host and sample sites (Figure 5). Multiple symbiont lineages, a trend in associating different cyanobacterial communities, were evident within the same sampling location.

### Mitochondrial Genealogy and Gene Variability of the Sponge *Hymeniacidon perlevis*

Analysis of the 658bp COI partial sequences identified five polymorphic sites, with four synonymous substitutions and one non-synonymous substitution within the 31 specimens of *H. perlevis* surveyed from the nine locations along the Atlantic coast of Portugal. The haplotype diversity (Hd) and nucleotide diversity (π) for the COI gene was 0.69+/−0.00518 and 0.00241+/−0.00034, respectively. Partial sequence derived from ATP6 (465bp) revealed two synonymous changes with Hd = 0.522+/−0.082 and π = 0.00177+/−0.00028. The spacer region (SP1) between COII and ATP6 amplified a product varying in size between ∼827 and 872 bp, with Hd = 0.533+/−0.081 and π = 0.00277+/−0.00039 (Table 1). We did not observe any tandem repeats in the SP1 regions as described previously in other demosponges species [42]. Two indels of 6 and 66 bp were detected among the SP1 sequenced specimens (see Figure S1).

**Table 1:** Diversity indices for the host sponge *H*. *perlevis*.

Mitochondrial Gene	Number of samples	Haplotype diversity (Hd-SD)	Nucleotide diversity (π-SD)
Cytochrome Oxidase I (COI)	n = 31	0.69+/−0.00518	0.00241+/−0.00034
ATP synthase subunit 6(ATP6)	n = 32	0.522+/−0.082	0.00177+/−0.00028
Spacer region(SP1)	n = 31	0.533+/−0.081	0.00277+/−0.00039

Haplotype (Hd) and nucleotide diversity (π) from COI, ATP6 and SP1 (Standard deviation SD).

**Figure 6:**
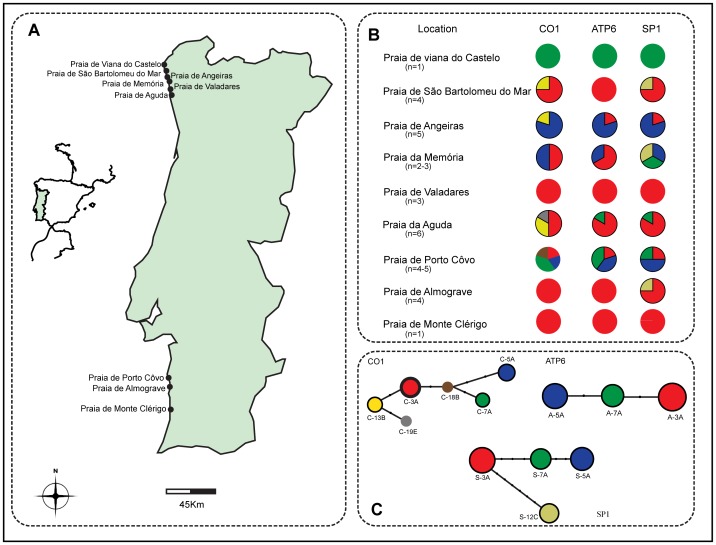
Geographical sampling locations of sponge host *H. perlevis* and phylogenetic network inferred from cytochrome oxidase I (COI), ATP synthase subunit 6 (ATP6) and spacer region (SP1). (A) Map indicating the specimen collection sites along the intertidal region of Atlantic coast of Portugal. (B) Pie chart represents frequency of haplotypes in each location with respective shading to the haplotypes. (C) The lower panel, networks from respective gene marker. Size of the circle is proportionate to the number of samples. The color for the circle is placed according to the haplotypes and the bold circles indicate the presence of haplotypes in more than one location. The black dots in the network indicate the number of mutation steps.

The median joining algorithm was used to estimate the mitochondrial genealogy [43] at intra-specific level. Reconstructed phylogenetic networks for the three different mitochondrial genes exhibited considerable branched genealogy among the sponge specimens sampled along the coast of Portugal (Figure 6). Six haplotypes of Cytochrome oxidase I DNA were obtained from 31 sequences, separated by up to nine mutational steps. The most abundant haplotype C-3A was shared by 16 specimens sampled from North and South of Portugal. The second most frequent haplotype C-5A was shared by six specimens from wide geographic locations. Other haplotypes, C-13B and C-7A, were shared among four and three sampling sites, respectively. Two singletons, C-19E and C-18B, were observed in two distinct sites (Table 2). Three haplotypes were observed within the ATP6 sequences (n = 32) and four within the SP1 sequences (n = 31) across different geographical locations (Figure 6).

**Table 2:** Number of haplotypes inferred from the mitochondrial gene markers COI, ATP6 and SP1.

			Haplotypes h = 6					
Gene	Sampling Location	Number of samples	*C-3A*	*C-5A*	*C-7A*	*C-18B*	*C-13B*	*C-19E*
	Praia de Monte Clérigo	1	1	–	–	–	–	–
	Praia de Almograve	4	4	–	–	–	–	–
	Praia de Porto Côvo	5	1	1	2	1	–	–
**CO1**	Praia de Valadares	3	3	–	–	–	–	–
	Praia da Aguda	6	3	–	–	–	2	1
	Praia da Memória	2	1	1	–	–	–	–
	Praia de São Bartolomeu do Mar	4	3	–	–	–	1	–
	Praia de Angeiras	5	–	4	–	–	1	–
	Praia de viana do castelo	1	–	–	1	–	–	–
			**Haplotypes h = 3**					
**Gene**	**Sampling Location**	**Number of samples**	***A-3A***	***A-5A***	***A-7A***			
	Praia de Monte Clérigo	1	1	–	–			
	Praia de Almograve	4	4	–	–			
	Praia de Porto Côvo	5	1	2	2			
**ATP6**	Praia de Valadares	3	3	–	–			
	Praia da Aguda	6	5	–	1			
	Praia da Memória	3	2	1	–			
	Praia de São Bartolomeu do Mar	4	4	–	–			
	Praia de Angeiras	5	1	4	–			
	Praia de viana do castelo	1	–	–	1			
			**Haplotypes h = 4**					
**Gene**	**Sampling Location**	**Number of samples**	***S-3A***	***S-5A***	***S-7A***	***S-12C***		
	Praia de Monte Clérigo	1	1	–	–	–		
	Praia de Almograve	4	3	–	–	1		
	Praia de Porto Côvo	4	1	2	1	–		
**SP1**	Praia de Valadares	3	3	–	–	–		
	Praia da Aguda	6	5	–	1	–		
	Praia da Memória	3	–	1	1	1		
	Praia de São Bartolomeu do Mar	4	3	–	–	1		
	Praia de Angeiras	5	1	4	–	–		
	Praia de viana do castelo	1	–	–	1	–		

Each table represents the number of haplotypes (h) for individual gene marker and its distribution in each sampling location. Derived haplotypes are represented in italics. Absence of sequence type is shows as a minus mark (−).

### Sponge-Cyanobacteria Cophylogeny

**Figure 7:**
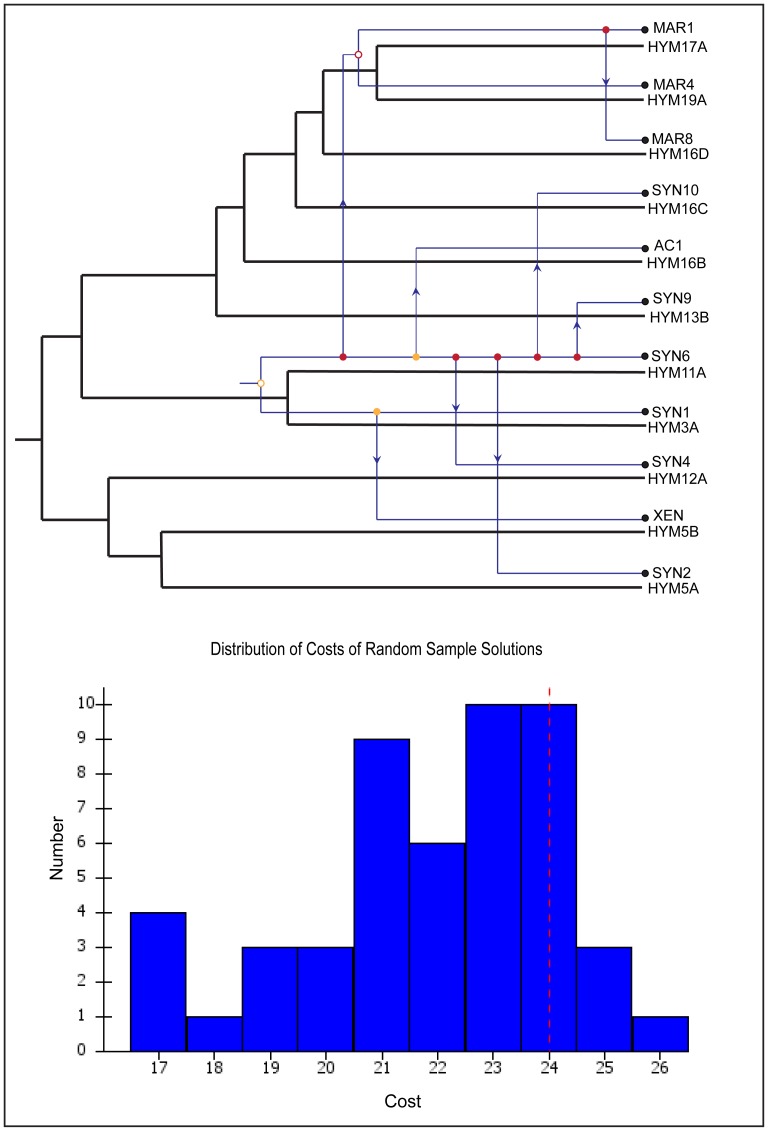
Coevolution and host switch between sponge host and its cyanobacterial symbionts. Host and symbiont tree are represented by black and blue mapped each other. Hollow dots depict coevolution, thick dots as duplication and arrow for host switch. The color orange and red of the node shows good and worst placement of the events. Uncultured marine cyanobacteria – MAR1, MAR4, MAR8; *Synechococcus* sp.- SYN1, SYN2, SYN4, SYN6, SYN9, SYN10; *Xenococcus* sp.- XEN; *Acaryochloris* sp.-AC1. Lower panel - histogram showing 30 random samples. Red line indicates original host-symbiont mapping and blue bars indicating random mapping.

Cophylogenetic analysis of the sponge host and the cyanobacterial symbionts revealed four cospeciation and eight host switch events (Figure 7). The switch of the associated uncultured marine cyanobacteria and *Synechococcus* sp. was frequently observed regardless of the different geographical locations. The statistical significance of the analysis did not support a coevolution pattern among the host and symbionts, as the majority of the random samples had lower cost than the original host-symbiont tip mapping (Figure 7).

## Discussion

### Cyanobacterial Diversity within *Hymeniacidon perlevis*

In this study, transmission electron microscopy and 16S rRNA sequencing identified the cyanobacteria *Xenococcus*-like morphotypes and *Acaryochloris* sp. in *H*. *perlevis*, which have never been reported to be sponge-associated cyanobacteria. The common sponge-associated cyanobacteria *Synechococcus* sp. [44] and other uncultured marine cyanobacteria from environmental samples were also detected among the intertidal sponge *H*. *perlevis* from different geographical locations along the Atlantic coast of Portugal.

We identified the *Xenococcus*-like morphotypes as a cyanobiont of the sponge host *H. perlevis* (sponge ID- HYM5B) from Praia de Porto Côvo (April 2010, the onset of spring season). Symbiotic association of *Xenococcus* sp. with metazoan species had previously been observed only in the tunic matrix of six species of ascidians from the cold temperate waters of Southern New Zealand [45]. The ultra-structural morphological features of the present cyanobacteria were very similar to that of the unicellular coccoid cyanobacteria *Xenococcus* sp. [46] and were further supported by 16S rRNA gene sequencing (sponge ID- HYM5B). During the same sampling period (April 2010) and in the same location (Praia de Porto Côvo, sponge collected just a meter apart), we found *Synechococcus* sp. to be the most predominant cyanobiont (sponge ID- HYM5A). Subsequent sampling from the same location in August and November 2010 (the onset of winter) found no evidence of the *Xenococcus* sp., but identified *Synechococcus* sp. (sponge ID-HYM10A) as the associated cyanobacteria. This suggests that *Xenococcus* sp. could maintain only a temporary or seasonal cyanobacteria-sponge association (since we observed it only during April 2010 in Praia de Porto Côvo). The host’s ability to acquire more than one strain of symbiotic cyanobacteria depending on the environmental conditions has been known among angiosperms *Gunnera* spp. which harbor the cyanobionts *Nostoc* spp. [47]. Similarly, acquisition of facultative microbes may influence the host’s reproduction and ecology as previously reported in crustaceans and insects [48]. We monitored a plausible variation in the morphological character (Figure 1) of the sampled sponge specimens mostly in the color and texture, during the collection in late winter in Praia de Porto Côvo. Different color morphs among sponge *Chondrilla australiensis* from Australian coast were believed to be an indication of different cyanobacterial symbionts [49], although later studies showed it was a response to photoacclimation by photobionts [23]. Taking into account the climatic variation and host’s possible life cycle during the sampling period, the presence of two symbionts from exactly at the same location (Praia de Porto Côvo) may suggest a temporary association for the host’s benefit. However, this phenomenon has not been observed in the samples from other geographical locations, where multiple symbionts coexisted at the time of sampling.

The cyanobacteria *Acaryochloris* sp., a common epiphyte of major macroalgae [50] and a symbiont of the colonial ascidians [51], was found in one of the sponges sampled from Praia de São Bartolomeu do Mar (sponge ID- HYM16B) (Figure 5). The oxygenic photoautotroph *Acaryochloris* sp. was widely detected as a symbiont in ascidians [52], [53]. Relatively high frequency of *Synechococcus* sp. and uncultured marine cyanobacteria throughout the sponges sampled suggested the co-existence of multiple symbiotic partners. Our data suggested there was a slight trend towards the association of symbionts that were specialist among the host across varied geographical locations, likely suggesting multiple associations over time [44]. Notwithstanding, species-specific (specialist) association of bacterial communities has been observed in the sister species *Hymeniacidon heliophila* across distant sampling locations [54]. The shifts of symbionts in filter feeder sponges should also be influenced by trophic changes, in particular for intertidal sponge such as *H*. *perlevis* that are submerged cyclically. Thus, the recruitment of diverse cyanobacteria species might be advantageous to sponge species that are constantly at risk of environmental exposure (*i.e*. drastic oxygen and radiation cyclic variations).

### *Hymeniacidon Perlevis* Genetic Variation

The spatial variation of the cyanobionts prompted us to further investigate the genetic diversity of the sponge host from different geographical locations in Portugal. Across the location of *H*. *perlevis* we identified three to six haplotypes at each of the three mitochondrial genes surveyed (ATP6, COI and SP1). Cytochrome oxidase I presented the higher level of haplotype diversity (Hd = 0.69+/−0.00518) across the nine localities compared to ATP6 and SP1. A low level of genetic variation at partial COI sequences (π = 0.0006) has been reported among *Crambe crambe* sponges separated by a geographic distance up to 3000 Km from the Mediterranean and the Atlantic coast [55]. Even less mitochondrial gene variability was observed among the sponge *Astrosclera willeyana sensu lato* (π = 0.00049) irrespective of the wide geographical coverage [56]. By contrast, our study showed a much higher genetic diversity for *H*. *perlevis* in a relatively restricted geographic range (∼500 Km) (ATP6, π = 0.00177; COI, π = 0.00241; and SP1, π = 0.00277) (Table 1) revealing considerable variability in this intertidal sponge species. Genetic richness of the studied *H. perlevis* sponge further suggests the involvement of sexual reproduction and pelagic larval dispersal [57], [58]. Considering the geographical distance of the sampling locations from North to South of Portugal (∼500 Km), we believe that environmental factors and reduced larval mobility [59] may be influencing the genetic makeup [60] of the intertidal marine sponge *H*. *perlevis*. Low dispersal ability of pelagic larvae and significant genetic diversity among localities was detected among the sponge *Hymeniacidon flavia* sampled along the Pacific coast of Japan using the mitochondrial gene NADH dehydrogenase subunit 5 [61], which could suggest similar explanation for higher genetic variability among *H. perlevis*.

### Host-symbiont co-phylogeny

Our characterization of the *H. perlevis* associated cyanobacteria and the host-symbiont cophylogenetic analysis provided insight about the spatial variation of the symbionts and host switching. Intra-specific host switching among symbionts of *H. perlevis* within and among geographical locations further validates the absence of any specificity of the symbionts, leaving unanswered whether the pattern is the result of colonization or coevolution of the symbiont in multiple lineage of sponge host [62]. Our cophylogenetic analysis suggests that different cyanobacterial symbionts switched the hosts at different sampling sites and time through either vertical or horizontal transmission.

### Conclusion

In this study, the characterization of cyanobionts in the most common intertidal to shallow subtidal marine sponge *Hymeniacidon perlevis* along the Atlantic coast of Portugal revealed the association of diverse photosymbionts. Electron microscopy and 16S rRNA gene sequence identified the presence of *Xenococcus*-like morphotypes. *Acaryochloris* sp., *Synechococcus sp.* and uncultured marine cyanobacteria were also harbored by specimens from different geographical locations, which suggest the change over time of the symbionts niche, probably favored by annual climatic cycles commonly observed in Praia de Porto Côvo. Variation in the preference of cyanobacteria within the host in different seasonal periods suggest that there is no relationship between geography and cyanobionts, and that there is a non-specialist association with the sponge host. Finally, relatively high intra-specific genetic variability within *H. perlevis* was identified with three mitochondrial markers (COI, ATP6 and SP1), which is consistent with reduced larval dispersal. The role of sexual reproduction and demography on the genetic makeup of *H. perlevis* has to be better studied with comprehensive sampling from wide geographical locations around the globe.

## Materials and Methods

### Sampling and Preservation

Specimens of the sponge *H. perlevis* (Montagu, 1818) were sampled from varied geographical locations of the Atlantic rocky shore of Portugal between March and November 2010 (Figure 6). No specific permits were required for the described field studies. The presence of this intertidal sponge in the South of Portugal is often reduced on these flatter beaches compared with the rocky beaches in the North. *Hymeniacidon perlevis* inhabit the rocky intertidal region and predominate in areas sheltered from the strong sun and tide, often lying at the base of the rocks. Sponge samples were collected in sterile plastic vessels in marine water, which keeps them stable during transportation at ambient temperature, and were later preserved in 70% ethanol for DNA extraction and morphological identification (e.g. ectosomal - choanosomal skeleton and spicule pattern evaluation).

### Transmission Electron Microscopy

Approximately 2 mm fresh sponge tissues was cut and immediately fixed in 2% glutaraldehyde in 50 mM sodium cacodylate buffer (pH 7.2) for 2 h, washed three times in double strength buffer, post-fixed with 2% osmium tetroxide in 50 mM sodium cacodylate buffer (pH 7.2) for 2 h, and washed again in double strength buffer. The dehydration was performed using an ethanol series (25–100%; v/v), and once using propylene oxide. Samples were embedded in mixtures of propylene oxide and Epon resin, followed by Epon for at least 24 h, before being placed in embedding moulds with Epon, and being allowed to polymerize at 55°C. Thin sections were cut with a Leica Reichert Supernova ultramicrotome, and mounted in copper grids. The sections were contrasted before being visualized using an electron microscope Zeiss EM C10 operating at 80 kV [63].

### Genomic DNA Extraction, Amplification and Sequencing

The sponge specimens were washed with sterile water before DNA extraction to ensure the complete removal of any free-living organisms and if necessary were examined by red autofluorescence on illumination with blue excitation using Olympus BX41 microscope. Total genomic DNA was extracted with a commercially available PureLink™ Genomic DNA Mini Kit (INVITROGEN), following the instructions of the manufacturer. Extracted genomic DNA was used for the amplification of cyanobacterial 16S ribosomal RNA (rRNA) and the sponge mitochondrial genes, namely the cytochrome oxidase subunit 1 (COI), ATP synthase subunit 6 (ATP6) and the spacer region (SP1). Reactions were performed in 50 µl volume, with 5 µl of 10× reaction buffer (BIOLINE), 5 µl of 2.5 mM DNTPs, (CITOMED), 2.5 µl of 2.5 mM MgCl_2_ (BIOLINE), 2.5 µl of 10 µm each primer, 0.1 µl of 5U/µl BIOTAQ DNA polymerase (BIOLINE) and 2.5 µl of 30 ng genomic DNA. Amplified products were directly purified or excised from gel using PureLink ™ Quick Gel Extraction and PCR Purification Combo Kit (INVITROGEN) and sequenced in both direction by the Macrogen Company (Seoul, South Korea) using an ABI 3730XL DNA Analyzer (Applied Biosystems).

### Sponge Associated Cyanobacterial 16S rRNA Gene Amplification

Two set of cyanobacteria specific primer pairs CYA359F (5′-GGGGAATYTTCCGCA ATGGG -3′) and CYA781R (5′-GACTACWGGGGTATCTAATCCCWT T -3′) [64] and 361F (5′-GAATTTTCCGCAATGGGC-3′) and 1459R (5′-GGTAAYGACTTCGGG CRT-3′) [65] were used to amplify two partial sequences of the 16S rRNA of 400 bp and 1000 bp, respectively, in 19 sponge samples (amplification was unsuccessful for some samples). PCR profile for both set of primers included an initial cycle (94°C for 4 min, 60°C for 2 min, 72°C for 2 min), then 30 cycles of 1 min at 94°C, 1 min at 60°C, 1 min at 72°C and a final extension time of 4 min at 72°C.

### Sponge Amplification of Partial COI, ATP6 and SP1

A partial fragment of the mitochondrial DNA COI gene was amplified with the degenerate primers used in the sponge barcoding project [66]. Primers dgLCO (5′-GGTCAACAAATCATAAAGAYATYG-3′) and dgHCO (5′-TAAACTTCA GGGTGACCAAARAAY-3′) were able to amplify 656 bp of COI fragment with the following PCR conditions: initial denaturing at 94°C for 2 min, followed by 35 cycles of (94°C for 40 s, 53°C for 40 s, 72°C for 1 min) and a final extension at 72°C for 10 min. ATP synthase 6 (ATP6) and SP1 were amplified using the specific primers: ATP6porF (5′- GTAGTCCAGGATAATT TAGG-3′)/ATP6porR (5′-GTTAATAGACAAAATACATAAG CCTG-3′) and CO2Fc (5′- GTKGCGCAAATCATTCWTTTATGC -3′)/ATP6R (5′-TGATCAAAATAWGCTGCTAA CAT -3′) [67]. The amplification parameters for ATP6 and spacer region were initiated at 94°C for 3 min, followed by 35 cycles of (93°C for 1 min, 48°C for 1 min, 72°C for 1 min) and a final extension at 72°C for 10 min.

### Data Analyses

Chromatograms from the sequencer were read and edited with FinchTV 1.4.0 (Geospiza, Inc.; Seattle, WA, USA; http://www.geospiza.com). The reliability of the cyanobacterial 16S rRNA gene sequences was confirmed using BLAST search (http://www.ncbi.nlm.nih.gov/BLAST/). Partial 16S rRNA gene sequences of varying length derived from two pair of primers were aligned with BioEdit Sequence Alignment Editor [68]. Ambiguous alignment regions were filtered using GBlocks [69], [70]. The sponge partial gene sequences of mitochondrial genes were also evaluated by BLAST search for their respective origin and similarity. COI, ATP6 and SP1 sequences were manually inspected for ambiguities, further aligned and edited in BioEdit. Intergenic regions were checked for the presence of repetitive sequences using Tandem Repeat Finder 4.04 program [71] and insertions/deletions were removed before population genetic analyses.

### Genetic Diversity and Haplotype Estimation

Basic population genetic parameters, genetic diversity indices, gene diversity and nucleotide diversity over loci [72] for host sponge sequences were estimated using DNAsp 5.10 [73]. Phylogenetic networks were constructed for the individual dataset derived from three mitochondrial gene sequences with the NETWORK 4.6.0.0 software (http://www.fluxus-engineering.com/) using the median-joining network algorithm [74]. Full median networks were calculated with the genetic distance parameter epsilon = 20 and Greedy FHP distance calculation method [75].

### Phylogenetic and PCoA Analyses

Total length ranging from ∼319 bp to 650 bp of the 16S rRNA gene sequence was generated using two pairs of cyanobacterial specific oligos, dataset comprised of symbionts from 19 host specimens. The sequences were manually edited for further analyses (see data analyses). A phylogenetic tree was constructed using the Neighbor-Joining method [76] implemented in MEGA5 [77] with Tajima-Nei substitution model [72] and a gamma distribution parameter estimated from the data. Node support was estimated using 1000 bootstrap replicates. Generated tree file and a file containing mapping ids of sponge associated cyanobacteria were uploaded to FastUniFrac [78] for Principal coordinates analysis (PCoA) with abundance-weighted option.

### Geophylogeny of Host and Cyanobionts

GenGIS [41], a geospatial information system, was used to draw a cyanobacterial 16S rRNA gene tree that connects the geographical sampling locations of the sponge host. NJ tree, digital map file (sampling location) and spatial co-ordinates of sample locations were used. Internal tree nodes were interpolated to the spatial location through a correlation line.

### Cophylogeny Analysis

Cophylogeny of the host and associated cyanobacterial community were evaluated with the software tool Jane [79]. Major events like coevolution, host switch and duplication were inferred by mapping the symbiont tree onto the host tree using a heuristic approach. The sequence dataset consisting of distinct symbiont from each location and its host mitochondrial gene was used for the analysis. The host tree was estimated with partially sequenced ATP6 mitochondrial gene and the cyanobacterial symbiont tree was inferred with the 16S rRNA gene sequence. The analysis was performed with default genetic algorithm parameters, producing best solution for the cophylogenetic events.

### Sequence Submission and Accession Numbers

Sequence data have been submitted to GenBank database under accession numbers for the respective gene markers under accession numbers, JX476996– JX477014 (16S rRNA), JX477015– JX477045 (COI), JX477046– JX 477077 (ATP6) and JX514032– JX514062 (SP1).

## Supporting Information

Figure S1 **Graphical view of alignment showing indels inferred from the spacer region located between COII and ATP6.** 32 specimens of sponge *H. perlevis* sequenced derived indels of 6 bp and 63 bp respectively. Indels are delimited by black rectangular box.
(DOCX)

Table S1 **Sponge specimen identification code from respective sampling location and the associated cyanobacteria.**
(DOCX)

## References

[1] DeLuca TH, Zackrisson O, Nilsson M-C, Sellstedt A (2002) Quantifying nitrogen-fixation in feather moss carpets of boreal forests. Nature 419: 917–920 doi:10.1038/nature01051. 12410308 10.1038/nature01051

[2] Read DJ, Duckett JG, Francis R, Ligrone R, Russell A (2000) Symbiotic fungal associations in “lower” land plants. Philosophical Transactions of the Royal Society of London Series B: Biological Sciences 355: 815–831 doi:10.1098/rstb.2000.0617. 10905611 10.1098/rstb.2000.0617 PMC1692782

[3] Heckman DS, Geiser DM, Eidell BR, Stauffer RL, Kardos NL, et al (2001) Molecular Evidence for the Early Colonization of Land by Fungi and Plants. Science 293: 1129–1133 doi:10.1126/science.1061457. 11498589 10.1126/science.1061457

[4] Lesser MP, Mazel CH, Gorbunov MY, Falkowski PG (2004) Discovery of Symbiotic Nitrogen-Fixing Cyanobacteria in Corals. Science 305: 997–1000 doi:10.1126/science.1099128. 15310901 10.1126/science.1099128

[5] Lee YK, Lee JH, Lee HK (2001) Microbial symbiosis in marine sponges. J Microbiol 39: 254–264.

[6] Ganot P, Moya A, Magnone V, Allemand D, Furla P, et al (2011) Adaptations to Endosymbiosis in a Cnidarian-Dinoflagellate Association: Differential Gene Expression and Specific Gene Duplications. PLoS Genet 7: e1002187 doi:10.1371/journal.pgen.1002187. 21811417 10.1371/journal.pgen.1002187 PMC3141003

[7] Yokobori S-I, Kurabayashi A, Neilan BA, Maruyama T, Hirose E (2006) Multiple origins of the ascidian-Prochloron symbiosis: molecular phylogeny of photosymbiotic and non-symbiotic colonial ascidians inferred from 18S rDNA sequences. Mol Phylogenet Evol 40: 8–19 doi:10.1016/j.ympev.2005.11.025. 16531073 10.1016/j.ympev.2005.11.025

[8] Wilkinson CR (1978) Microbial associations in sponges. I. Ecology, physiology and microbial populations of coral reef sponges. Mar Biol 49: 161–167 doi:10.1007/BF00387115.

[9] Wilkinson CR (1984) Immunological Evidence for the Precambrian Origin of Bacterial Symbioses in Marine Sponges. Proceedings of the Royal Society of London Series B Biological Sciences 220: 509–518 doi:10.1098/rspb.1984.0017.

[10] Wilkinson C (1987) Significance of microbial symbionts in sponge evolution and ecology. Symbiosis 4: 135–145.

[11] Sarà M, Bavestrello G, Cattaneo-vietti R, Cerrano C (1998) Endosymbiosis in sponges: Relevance for epigenesis and evolution. Symbiosis 25: 57–70.

[12] O’Brien HE, Miadlikowska J, Lutzoni F (2005) Assessing host specialization in symbiotic cyanobacteria associated with four closely related species of the lichen fungus Peltigera. European Journal of Phycology 40: 363 doi:10.1080/09670260500342647.

[13] Usher KM (2008) The ecology and phylogeny of cyanobacterial symbionts in sponges. Marine Ecology 29: 178–192 doi:10.1111/j.1439-0485.2008.00245.x. 10.1007/s00248-003-1062-3 15546037

[14] Carballo JL, vila E (2004) Population dynamics of a mutualistic interaction between the sponge Haliclona caerulea and the red alga Jania adherens. Mar Ecol Prog Ser 279: 93–104 doi:10.3354/meps279093.

[15] Wilkinson CR, Fay P (1979) Nitrogen fixation in coral reef sponges with symbiotic cyanobacteria. Nature 279: 527–529 doi:10.1038/279527a0.

[16] Shick JM, Dunlap WC (2002) MYCOSPORIN-LIKE AMINOACIDS AND RELATED GADUSOLS?: Biosynthesis, Accumulation, and UV-Protective Functions in Aquatic Organisms. Annu Rev Physiol 64: 223–262 doi:10.1146/annurev.physiol.64.081501.155802. 11826269 10.1146/annurev.physiol.64.081501.155802

[17] Proteau PJ, Gerwick WH, Garcia-Pichel F, Castenholz R (1993) The structure of scytonemin, an ultraviolet sunscreen pigment from the sheaths of cyanobacteria. Experientia 49: 825–829. 8405307 10.1007/BF01923559

[18] Cox PA, Banack SA, Murch SJ, Rasmussen U, Tien G, et al (2005) Diverse taxa of cyanobacteria produce beta-N-methylamino-L-alanine, a neurotoxic amino acid. Proc Natl Acad Sci USA 102: 5074–5078 doi:10.1073/pnas.0501526102. 15809446 10.1073/pnas.0501526102 PMC555964

[19] Cheshire AC, Wilkinson CR, Seddon S, Westphalen G (1997) Bathymetric and seasonal changes in photosynthesis and respiration of the phototrophic sponge Phyllospongia lamellosa in comparison with respiration by the heterotrophic sponge Ianthella basta on Davies Reef, Great Barrier Reef. Mar Freshwater Res 48: 589–599.

[20] Maldonado M, Young CM (1998) Limits on the bathymetric distribution of keratose sponges: a field test in deep water. Available:http://digital.csic.es/handle/10261/3243?mode=full&submit_simple=Showfullitemrecord. Accessed 7 August 2012.

[21] Larkum AWD, Kennedy IR, Muller WJ (1988) Nitrogen fixation on a coral reef. Marine Biology 98: 143–155 doi:10.1007/BF00392669.

[22] Feldmann J (1935) Sur quelques cyanophycées vivant dans le tissu des éponges de banyules.Second volume jubilaire Tome 75, p. 331 à 404. 1er Décembre 1933. Archives de zoologie expérimentale et générale 75: 381.

[23] Usher KM, Fromont J, Sutton DC, Toze S (2004) The biogeography and phylogeny of unicellular cyanobacterial symbionts in sponges from Australia and the Mediterranean. Microb Ecol 48: 167–177 doi:10.1007/s00248-003-1062-3. 15546037 10.1007/s00248-003-1062-3

[24] Larkum L (1999) The cyanobacteria of coral reefs. Marine Cyanobacteria. In: Charpy, L, Larkum, AWD (Eds.). Bull I’Inst oceanograph Monaco Num special 19 Monaco Musee Oceanograph, 149–167.

[25] Berthold RJ, Borowitzka MA, Mackay MA (1982) The ultrastructure of Oscillatoria spongeliae, the blue-green algal endosymbiont of the sponge Dysidea herbacea. Phycologia 21: 327–335 doi:10.2216/i0031-8884-21-3-327.1.

[26] Ridley CP, John Faulkner D, Haygood MG (2005) Investigation of Oscillatoria spongeliae-Dominated Bacterial Communities in Four Dictyoceratid Sponges. Appl Environ Microbiol 71: 7366–7375 doi:10.1128/AEM.71.11.7366-7375.2005. 16269779 10.1128/AEM.71.11.7366-7375.2005 PMC1287642

[27] Thacker RW, Starnes S (2003) Host specificity of the symbiotic cyanobacterium Oscillatoria spongeliae in marine sponges,Dysidea spp. Marine Biology 142: 643–648.

[28] Erwin PM, Thacker RW (2008) Cryptic diversity of the symbiotic cyanobacterium Synechococcus spongiarum among sponge hosts. Molecular Ecology 17: 2937–2947 doi:10.1111/j.1365-294X.2008.03808.x. 18489545 10.1111/j.1365-294X.2008.03808.x

[29] Hentschel U, Hopke J, Horn M, Friedrich AB, Wagner M, et al (2002) Molecular Evidence for a Uniform Microbial Community in Sponges from Different Oceans. Appl Environ Microbiol 68: 4431–4440 doi:10.1128/AEM.68.9.4431-4440.2002. 12200297 10.1128/AEM.68.9.4431-4440.2002 PMC124103

[30] Usher KM, Bergman B, Raven JA (2007) Exploring Cyanobacterial Mutualisms. Annu Rev Ecol Evol Syst 38: 255–273 doi:10.1146/annurev.ecolsys.38.091206.095641.

[31] Schwarz JA, Krupp DA, Weis VM (1999) Late Larval Development and Onset of Symbiosis in the Scleractinian Coral Fungia scutaria. Biol Bull 196: 70–79. 25575388 10.2307/1543169

[32] Avise JC, Arnold J, Ball RM, Bermingham E, Lamb T, et al (1987) Intraspecific Phylogeography: The Mitochondrial DNA Bridge Between Population Genetics and Systematics. Annual Review of Ecology and Systematics 18: 489–522 doi:10.1146/annurev.es.18.110187.002421.

[33] DeBiasse MB, Richards VP, Shivji MS (2010) Genetic assessment of connectivity in the common reef sponge, Callyspongia vaginalis (Demospongiae: Haplosclerida) reveals high population structure along the Florida reef tract. Coral Reefs 29: 47–55 doi:10.1007/s00338-009-0554-0.

[34] Xavier JR, Rachello-Dolmen PG, Parra-Velandia F, Schönberg CHL, Breeuwer JAJ, et al (2010) Molecular evidence of cryptic speciation in the “cosmopolitan” excavating sponge Cliona celata (Porifera, Clionaidae). Mol Phylogenet Evol 56: 13–20 doi:10.1016/j.ympev.2010.03.030. 20363344 10.1016/j.ympev.2010.03.030

[35] Redmond NE, Raleigh J, van Soest RWM, Kelly M, Travers SAA, et al (2011) Phylogenetic Relationships of the Marine Haplosclerida (Phylum Porifera) Employing Ribosomal (28S rRNA) and Mitochondrial (cox1, nad1) Gene Sequence Data. PLoS ONE 6: e24344 doi:10.1371/journal.pone.0024344. 21931685 10.1371/journal.pone.0024344 PMC3172223

[36] Cabioch L (1968) Contribution ą la connaissance de la faune des spongiaires de la Manche occidentale: démosponges de la région de Roscoff. Cahiers de Biologie marine.

[37] Zhang H, Lee YK, Zhang W, Lee HK (2006) Culturable actinobacteria from the marine sponge Hymeniacidon perleve: isolation and phylogenetic diversity by 16S rRNA gene-RFLP analysis. Antonie Van Leeuwenhoek 90: 159–169 doi:10.1007/s10482-006-9070-1. 16871424 10.1007/s10482-006-9070-1

[38] Xin Y, Huang J, Deng M, Zhang W (2008) Culture-independent nested PCR method reveals high diversity of actinobacteria associated with the marine sponges Hymeniacidon perleve and Sponge sp. Antonie Van Leeuwenhoek 94: 533–542 doi:10.1007/s10482-008-9270-y. 18670903 10.1007/s10482-008-9270-y

[39] Jin Y, Guo P, Sun L, Yu X, Zhang W (2006) Phylogenetic diversity of endocellular bacteria marine sponge Hymeniacidon perleve. Wei Sheng Wu Xue Bao 46: 875–878. 17302146

[40] Ackers RG., Moss D, Picton B. (1992) Sponges of the British Isles (’Sponge V’), a colour guide and working document.

[41] Parks DH, Porter M, Churcher S, Wang S, Blouin C, et al (2009) GenGIS: A geospatial information system for genomic data. Genome Res 19: 1896–1904 doi:10.1101/gr.095612.109. 19635847 10.1101/gr.095612.109 PMC2765287

[42] Wang X, Lavrov DV (2008) Seventeen New Complete mtDNA Sequences Reveal Extensive Mitochondrial Genome Evolution within the Demospongiae. PLoS ONE 3: e2723 doi:10.1371/journal.pone.0002723. 18628961 10.1371/journal.pone.0002723 PMC2444032

[43] Cassens I, Mardulyn P, Milinkovitch MC (2005) Evaluating Intraspecific “Network” Construction Methods Using Simulated Sequence Data: Do Existing Algorithms Outperform the Global Maximum Parsimony Approach? Systematic Biology 54: 363–372 doi:10.1080/10635150590945377. 16012104 10.1080/10635150590945377

[44] Steindler L, Huchon D, Avni A, Ilan M (2005) 16S rRNA phylogeny of sponge-associated cyanobacteria. Appl Environ Microbiol 71: 4127–4131 doi:10.1128/AEM.71.7.4127-4131.2005. 16000832 10.1128/AEM.71.7.4127-4131.2005 PMC1168989

[45] Lambert G, Lambert CC, Waaland JR (1996) Algal Symbionts in the Tunics of Six New Zealand Ascidians (Chordata, Ascidiacea). Invertebrate Biology 115: 67–78 doi:10.2307/3226942.

[46] Montejano G, Komarek J (1994) Goldmorgan (1994) Fresh-Water Epiphytic Cyanoprokaryotes from Central Mexico.2. Heterogeneity of the Genus Xenococcus. Archiv Fur Protistenkunde 144: 383–405.

[47] Wilmotte A (2004) Molecular Evolution and Taxonomy of the Cyanobacteria. In: Bryant DA, editor. The Molecular Biology of Cyanobacteria. Dordrecht: Kluwer Academic Publishers, Vol. 1. 1–25. Available:http://www.springerlink.com/content/jw7w22r10l477720/. Accessed 24 September 2011.

[48] Engelstädter J, Hurst GDD (2009) The Ecology and Evolution of Microbes that Manipulate Host Reproduction. Annu Rev Ecol Evol Syst 40: 127–149 doi:10.1146/annurev.ecolsys.110308.120206.

[49] Fromont J (1999) Reproduction of some demosponges in a temperate Australian shallow water habitat. MEMOIRS-QUEENSLAND MUSEUM 44: 185–192.

[50] Ohkubo S, Miyashita H, Murakami A, Takeyama H, Tsuchiya T, et al (2006) Molecular Detection of Epiphytic Acaryochloris spp. on Marine Macroalgae. Appl Environ Microbiol 72: 7912–7915 doi:10.1128/AEM.01148-06. 17028237 10.1128/AEM.01148-06 PMC1694208

[51] Miyashita H, Ikemoto H, Kurano N, Adachi K, Chihara M, et al (1996) Chlorophyll d as a major pigment. Nature 383: 402 doi:10.1038/383402a0.

[52] Kuhl M, Chen M, Ralph PJ, Schreiber U, Larkum AWD (2005) Ecology: A niche for cyanobacteria containing chlorophyll d. Nature 433: 820 doi:10.1038/433820a. 15729331 10.1038/433820a

[53] López-Legentil S, Song B, Bosch M, Pawlik JR, Turon X (2011) Cyanobacterial Diversity and a New Acaryochloris-Like Symbiont from Bahamian Sea-Squirts. PLoS ONE 6: e23938 doi:10.1371/journal.pone.0023938. 21915246 10.1371/journal.pone.0023938 PMC3161822

[54] Erwin PM, Olson JB, Thacker RW (2011) Phylogenetic diversity, host-specificity and community profiling of sponge-associated bacteria in the northern Gulf of Mexico. PLoS ONE 6: e26806 doi:10.1371/journal.pone.0026806. 22073197 10.1371/journal.pone.0026806 PMC3206846

[55] Duran S, Pascual M, Turon X (2004) Low levels of genetic variation in mtDNA sequences over the western Mediterranean and Atlantic range of the sponge Crambe crambe (Poecilosclerida). Marine Biology 144: 31–35 doi:10.1007/s00227-003-1178-5.

[56] Wörheide G (2006) Low variation in partial cytochrome oxidase subunit I (COI) mitochondrial sequences in the coralline demosponge Astrosclera willeyana across the Indo-Pacific. Marine Biology 148: 907–912 doi:10.1007/s00227-005-0134-y.

[57] Stone AR (2009) Growth and reproduction of Hymeniacidon perleve (Montagu) (Porifera) in Langstone Harbour Hampshire. Journal of Zoology 161: 443–459 doi:10.1111/j.1469-7998.1970.tb02048.x.

[58] Gaino E, Frine C, Giuseppe C (2010) Reproduction of the Intertidal Sponge Hymeniacidon perlevis (Montagu) Along a Bathymetric Gradient. The Open Marine Biology Journal 4: 47–56.

[59] Manuel M, Craig Y (1996) Effects of physical factors on larval behavior, settlement and recruitment of four tropical demosponges. Mar Ecol Prog Ser 138: 169–180 doi:10.3354/meps138169.

[60] Ayre DJ, Hughes TP (2004) Climate change, genotypic diversity and gene flow in reef-building corals. Ecology Letters 7: 273–278 doi:10.1111/j.1461-0248.2004.00585.x.

[61] Hoshino S, Saito DS, Fujita T (2008) Contrasting genetic structure of two Pacific Hymeniacidon species. Hydrobiologia 603: 313–326 doi:10.1007/s10750-008-9295-2.

[62] Brooks DR, McLennan DA (1991) Phylogeny, Ecology, and Behavior: A Research Program in Comparative Biology. 1st ed. University Of Chicago Press. 441 p.

[63] Seabra R, Santos A, Pereira S, Moradas-Ferreira P, Tamagnini P (2009) Immunolocalization of the uptake hydrogenase in the marine cyanobacterium Lyngbya majuscula CCAP 1446/4 and two Nostoc strains. FEMS Microbiol Lett 292: 57–62 doi:10.1111/j.1574-6968.2008.01471.x. 19222582 10.1111/j.1574-6968.2008.01471.x

[64] Nübel U, Garcia-Pichel F, Muyzer G (1997) PCR primers to amplify 16S rRNA genes from cyanobacteria. Appl Environ Microbiol 63: 3327–3332. 9251225 10.1128/aem.63.8.3327-3332.1997 PMC168636

[65] Diaz MC (1997) Molecular detection and characterization of specific bacterial groups associated with tropical sponges. Proceedings of the 8th International Coral Reef Symposium, Panama. Vol. 24: 1399–1402.

[66] Meyer CP, Geller JB, Paulay G (2005) Fine scale endemism on coral reefs: archipelagic differentiation in turbinid gastropods. Evolution 59: 113–125. 15792232

[67] Rua CPJ, Zilberberg C, Sole-Cava AM (2011) New Polymorphic Mitochondrial Markers for Sponge Phylogeography. Journal of the Marine Biological Association of the United Kingdom FirstView: 1–8. doi:10.1017/S0025315410002122.

[68] Hall T (1999) BioEdit: a user-friendly biological sequence alignment editor and analysis program for Windows 95/98/NT. Nucleic Acids Symposium Series 41: 95–98.

[69] Castresana J (2000) Selection of conserved blocks from multiple alignments for their use in phylogenetic analysis. Mol Biol Evol 17: 540–552. 10742046 10.1093/oxfordjournals.molbev.a026334

[70] Talavera G, Castresana J (2007) Improvement of phylogenies after removing divergent and ambiguously aligned blocks from protein sequence alignments. Syst Biol 56: 564–577 doi:10.1080/10635150701472164. 17654362 10.1080/10635150701472164

[71] Benson G (1999) Tandem repeats finder: a program to analyze DNA sequences. Nucleic Acids Res 27: 573–580. 9862982 10.1093/nar/27.2.573 PMC148217

[72] Tajima F (1983) Evolutionary relationship of DNA sequences in finite populations. Genetics 105: 437–460. 6628982 10.1093/genetics/105.2.437 PMC1202167

[73] Librado P, Rozas J (2009) DnaSP v5: a software for comprehensive analysis of DNA polymorphism data. Bioinformatics 25: 1451–1452 doi:10.1093/bioinformatics/btp187. 19346325 10.1093/bioinformatics/btp187

[74] Bandelt HJ, Forster P, Röhl A (1999) Median-joining networks for inferring intraspecific phylogenies. Molecular Biology and Evolution 16: 37–48. 10331250 10.1093/oxfordjournals.molbev.a026036

[75] Foulds LR, Hendy MD, Penny D (1979) A graph theoretic approach to the development of minimal phylogenetic trees. J Mol Evol 13: 127–149. 480370 10.1007/BF01732868

[76] Saitou N, Nei M (1987) The neighbor-joining method: a new method for reconstructing phylogenetic trees. Mol Biol Evol 4: 406–425. 3447015 10.1093/oxfordjournals.molbev.a040454

[77] Tamura K, Peterson D, Peterson N, Stecher G, Nei M, et al (2011) MEGA5: molecular evolutionary genetics analysis using maximum likelihood, evolutionary distance, and maximum parsimony methods. Mol Biol Evol 28: 2731–2739 doi:10.1093/molbev/msr121. 21546353 10.1093/molbev/msr121 PMC3203626

[78] Hamady M, Lozupone C, Knight R (2010) Fast UniFrac: facilitating high-throughput phylogenetic analyses of microbial communities including analysis of pyrosequencing and PhyloChip data. ISME J 4: 17–27 doi:10.1038/ismej.2009.97. 19710709 10.1038/ismej.2009.97 PMC2797552

[79] Conow C, Fielder D, Ovadia Y, Libeskind-Hadas R (2010) Jane: a new tool for the cophylogeny reconstruction problem. Algorithms Mol Biol 5: 16–16 doi:10.1186/1748-7188-5-16. 20181081 10.1186/1748-7188-5-16 PMC2830923

