# Supplementary material for: Unusual Symbiotic Cyanobacteria Association in the Genetically Diverse Intertidal Marine Sponge Hymeniacidon perlevis (Demospongiae, Halichondrida)
Source: PLoS One. 2012 Dec 14;7(12):e51834. doi: 10.1371/journal.pone.0051834 (PMC3522618; doi:10.1371/journal.pone.0051834)
Supplement: Figure S1 — Graphical view of alignment showing indels inferred from the spacer region located between COII and ATP6. 32 specimens of sponge H. perlevis sequenced derived indels of 6 bp and 63 bp respectively. Indels are delimited by black rectangular box. (DOCX) [file pone.0051834.s001.docx]

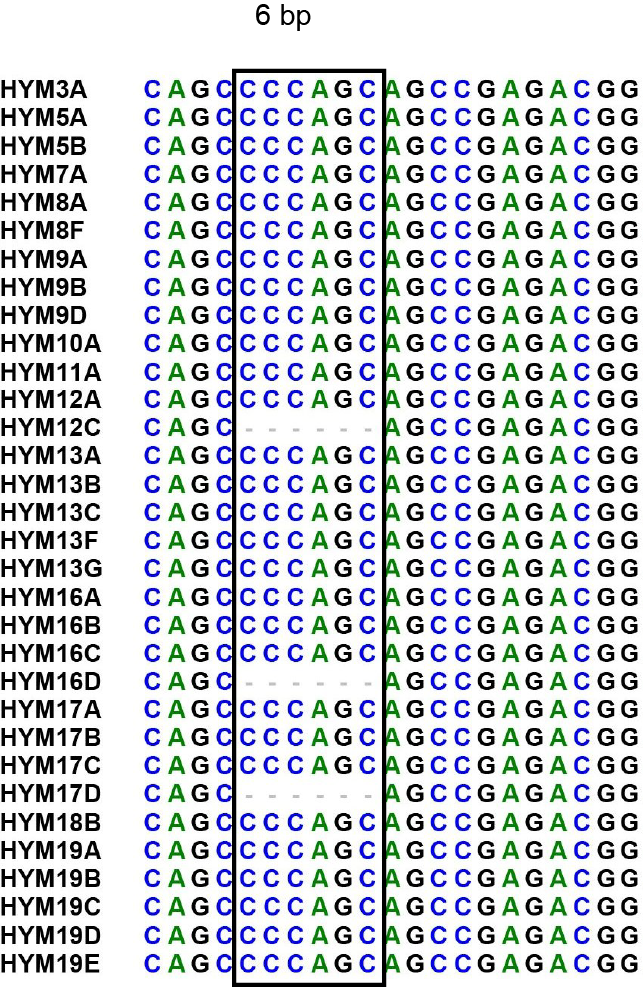


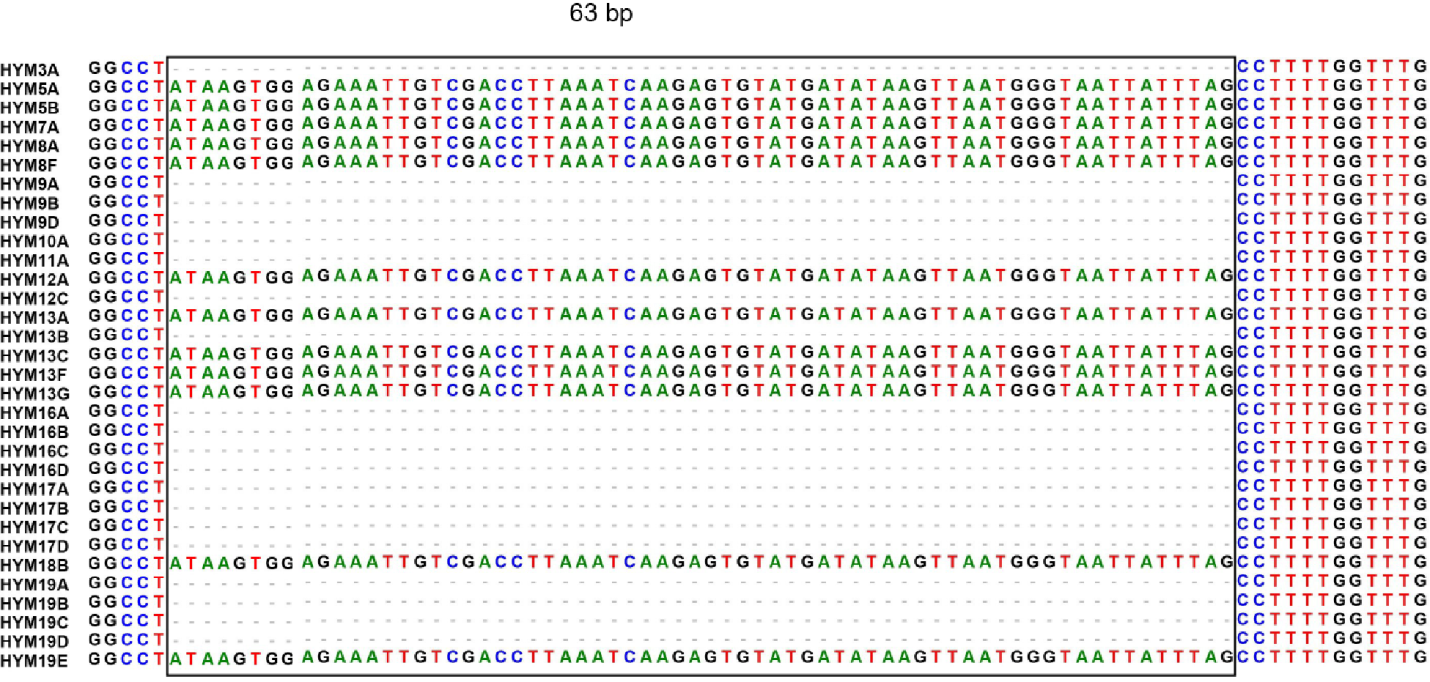


**Figure S1. Indels inferred from the spacer region located between COII and ATP6.**  32 specimens of sponge *H. perlevis* sequenced derived indels of 6 bp and 63 bp respectively. Indels are delimited by black rectangular box.
