# Supplementary material for: Unusual Symbiotic Cyanobacteria Association in the Genetically Diverse Intertidal Marine Sponge Hymeniacidon perlevis (Demospongiae, Halichondrida)
Source: PLoS One. 2012 Dec 14;7(12):e51834. doi: 10.1371/journal.pone.0051834 (PMC3522618; doi:10.1371/journal.pone.0051834)
Supplement: Table S1 — Sponge specimen identification code from respective sampling location and the associated cyanobacteria. (DOCX) [file pone.0051834.s002.docx]

**Table S1. Sponge specimen identification code from respective sampling location and the associated cyanobacteria.**

| Sponge Specimen ID | Sampling site | Inferred cyanobionts and its ID |
| --- | --- | --- |
| HYM3A | Praia da Monte Clérigo | *Synechococcus* sp. (SYN1) |
| HYM5A | Praia de Porto Côvo | *Synechococcus* sp. (SYN2) |
| HYM5B | Praia de Porto Côvo | *Xenococcu*s sp. (XEN) |
| HYM10A | Praia de Porto Côvo | *Synechococcus* sp. (SYN3) |
| HYM11A | Praia da Aguda | *Synechococcus* sp. (SYN6) |
| HYM12A | Praia da Memória | *Synechococcus* sp. (SYN4) |
| HYM13B | Praia de Angeiras | *Synechococcus* sp. (SYN9) |
| HYM16B | Praia de São Bartolomeu do Mar | *Acaryochloris* sp. (AC1) |
| HYM16C | Praia de São Bartolomeu do Mar | *Synechococcus* sp. SYN10) |
| HYM16D | Praia de São Bartolomeu do Mar | Uncultured marine cyanobacterium (MAR8) |
| HYM17A | Praia de Almograve | Uncultured marine cyanobacterium (MAR1) |
| HYM17C | Praia de Almograve | Uncultured marine cyanobacterium (MAR2) |
| HYM17D | Praia de Almograve | Uncultured marine cyanobacterium (MAR3) |
| HYM19A | Praia da Aguda | Uncultured marine cyanobacterium (MAR4) |
| HYM19B | Praia da Aguda | Uncultured marine cyanobacterium (MAR5) |
| HYM19C | Praia da Aguda | Uncultured marine cyanobacterium (MAR7) |
| HYM19D | Praia da Aguda | Uncultured marine cyanobacterium (MAR6) |
| HYM19F | Praia da Aguda | *Synechococcus* sp. (SYN5) |
| HYM19G | Praia da Aguda | *Synechococcus* sp. (SYN8) |
